# Diagnostic delay is associated with uveitis and inflammatory bowel disease in AS: a study of extra-musculoskeletal manifestations in SpA

**DOI:** 10.1093/rheumatology/kead225

**Published:** 2023-05-15

**Authors:** Xabier Michelena, Sizheng Steven Zhao, Carla Marco-Pascual, Miriam Almirall, Eduardo Collantes-Estevez, Pilar Font-Ugalde, Clementina López-Medina, James Cheng-Chung Wei, Ann W Morgan, Jesús Rodríguez, Xavier Juanola, Janitzia Vázquez-Mellado, Helena Marzo-Ortega

**Affiliations:** Rheumatology Unit, Vall d’Hebron Hospital Universitari, Vall d’Hebron Barcelona Hospital Campus, Barcelona, Spain; NIHR Leeds BRC, Leeds Teaching Hospitals NHS Trust and School of Medicine, University of Leeds, Leeds, UK; Centre for Epidemiology Versus Arthritis, Division of Musculoskeletal and Dermatological Science, The University of Manchester, Manchester, UK; Rheumatology Unit, Hospital Dos de Maig—Consorci Sanitari Integral, Barcelona, Spain; Rheumatology Unit, Bellvitge University Hospital, L’Hospitalet de Llobregat, Spain; NIHR Leeds BRC, Leeds Teaching Hospitals NHS Trust and School of Medicine, University of Leeds, Leeds, UK; Rheumatology Unit, Reina Sofia University Hospital and Maimonides Institute for Research in Biomedicine of Cordoba (IMIBIC), University of Córdoba, Córdoba, Spain; Rheumatology Unit, Reina Sofia University Hospital and Maimonides Institute for Research in Biomedicine of Cordoba (IMIBIC), University of Córdoba, Córdoba, Spain; Rheumatology Unit, Reina Sofia University Hospital and Maimonides Institute for Research in Biomedicine of Cordoba (IMIBIC), University of Córdoba, Córdoba, Spain; Division of Allergy, Immunology and Rheumatology, Department of Internal Medicine, Chung Shan Medical University Hospital, Taichung, Taiwan; NIHR Leeds BRC, Leeds Teaching Hospitals NHS Trust and School of Medicine, University of Leeds, Leeds, UK; Rheumatology Unit, Bellvitge University Hospital, L’Hospitalet de Llobregat, Spain; Rheumatology Unit, Bellvitge University Hospital, L’Hospitalet de Llobregat, Spain; Rheumatology Unit, Hospital General de Mexico, Mexico City, Mexico; NIHR Leeds BRC, Leeds Teaching Hospitals NHS Trust and School of Medicine, University of Leeds, Leeds, UK

**Keywords:** AS, PsA, psoriasis, uveitis, IBD, diagnostic delay

## Abstract

**Objectives:**

To examine the prevalence of extra-musculoskeletal manifestations (EMM) and the association between diagnostic delay and their incidence in AS and PsA.

**Methods:**

This was a retrospective, cohort study comprising two single centre cohorts in Europe and one multicentre cohort in Latin America (RESPONDIA). Crude prevalence of EMMs (uveitis, IBD and psoriasis) was calculated across geographic area and adjusted by direct standardization. Cox proportional hazard analysis was performed to assess the association between diagnostic delay and EMM incidence.

**Results:**

Of 3553 patients, 2097 had AS and 1456 had PsA. The overall prevalence of uveitis was 22.9% (95% CI: 21.1, 24.8) in AS and 3.8% (95% CI: 2.9, 5.0) in PsA; 8.1% (95% CI: 7.0, 9.4) and 2.1% (1.3, 2.9), respectively, for IBD; and 11.0% (95% CI: 9.7, 12.4) and 94.6% (93.0, 95.9), respectively, for psoriasis. The EMM often presented before the arthritis (uveitis 45.1% and 33.3%, and IBD 37.4% and 70%, in AS and PsA, respectively). In the multivariable model, longer diagnostic delay (≥5 years) associated with more uveitis (hazard ratio [HR] 4.01; 95% CI: 3.23, 4.07) and IBD events (HR 1.85; 95% CI: 1.28, 2.67) in AS. Diagnostic delay was not significantly associated with uveitis (HR 1.57; 95% CI: 0.69, 3.59) or IBD events (HR 1.59; 95% CI: 0.39, 6.37) in PsA.

**Conclusion:**

EMMs are more prevalent in AS than PsA and often present before the onset of the articular disease. A longer diagnostic delay is associated with the ‘*de novo*’ appearance of uveitis and IBD in AS, highlighting the need to enhance diagnostic strategies to shorten the time from first symptom to diagnosis in SpA.

Rheumatology key messages

- The prevalence of uveitis and IBD is higher in AS when compared with PsA.
- Uveitis or IBD may present before the onset of the SpA diagnosis in a significant proportion of people with AS and PsA.
- A longer diagnostic delay is associated with a higher probability of uveitis and IBD in AS.

## Introduction

Axial spondyloarthritis (axSpA) encompassing AS, also known as radiographic axSpA (r-axSpA), and PsA are two distinct chronic inflammatory diseases under the umbrella of the spondyloarthritides (SpA) [1, 2]. The different SpAs share common clinical and genetic characteristics including inflammation of axial (spine/sacroiliac) and peripheral joints with extra-musculoskeletal manifestations (EMMs). EMMs strongly linked to the SpA disease group are uveitis, IBD and psoriasis [3]. Their prevalence has been outlined in recent meta-analyses as higher in axSpA (pooled prevalence of uveitis, IBD and psoriasis of 23%, 6.4% and 10.2%, respectively) than PsA (pooled prevalence of 3.2% for uveitis and 3.3% IBD of 3.2%) [4, 5].

In axSpA, EMMs have been shown to impact the disease course with a recent report revealing increased cardiovascular risk that appears proportional to the number of EMMs in addition to higher axSpA disease activity and functional impairment [6, 7]. By contrast, this relationship with functional disability was not seen in the OASIS cohort after 12 years of follow-up [8]. The relationship between disease duration and EMM incidence has been confirmed in several studies [8–11]. Also, HLA-B27 positivity has been shown to be associated with uveitis in several axSpA cohorts [11, 12].

Despite greater awareness and improved diagnostic techniques over the last two decades, delay to diagnosis remains a significant unmet need in SpA when compared with other chronic inflammatory arthritides. This is particularly the case in axSpA with consequent impact on disease outcome [13]. Longer diagnostic delay may also lead to higher incidence of EMMs, a hypothesis that has not been fully investigated yet. The aims of this study are to examine the prevalence of EMM and the effect of diagnostic delay in their appearance in large cohorts of AS and PsA.

## Methods

We performed a retrospective, multicentre, cohort study across three geographic areas comprising two single centre cohorts in Europe (Chapel Allerton Hospital, Leeds, UK and Hospital Universitari de Bellvitge, Barcelona, Spain) and one multicentre cohort in Latin America (RESPONDIA Cohort comprising Argentina, Brazil, Chile, Costa Rica, Mexico, Peru, Uruguay, Venezuela) [14]. Data from patients older than 18 years with a primary clinician diagnosis of AS or PsA were included in the analyses. All participants provided informed written consent and the study was approved by the Hospital Universitari de Bellvitge Research Ethics Committee (Approval Number PR004/20). Individual cohort ethics and inclusion criteria details are shown in Supplementary Materials, available at *Rheumatology* online. Protocol design in each cohort predated the development of ASAS and CASPAR classification criteria, and hence the original nomenclature utilized in the different studies referring to ankylosing spondylitis rather than r-axSpA will be utilized in this report.

Age, sex, date of onset of musculoskeletal symptoms, date of diagnosis, date of last follow-up, disease duration (calculated as time from diagnosis to last available follow-up date), HLA-B27 status, history of uveitis, IBD (including Crohn’s disease, ulcerative colitis and undifferentiated IBD) and psoriasis (as confirmed by a physician) together with their onset dates were collected as variables. Data from all cohorts were collected at the inclusion time point with missing values retrieved from clinical notes at a later time point.

### Statistical analysis

Demographic characteristics were compared by disease (AS and PsA) and across the three geographic cohorts. Student’s *t* or Mann–Whitney *U* tests for continuous variables and the chi-square test for categorical variables were used as appropriate. Prevalence of EMM was calculated (with 95% CI) overall and for each of the three geographic areas and by disease. Crude prevalence was adjusted for age by direct standardization using the WHO World standard population as reference [15].

Diagnostic delay was defined as the time between date of musculoskeletal symptom onset and date of diagnosis. To examine the relationship between diagnostic delay and appearance of EMM, only EMMs that occurred after the date of diagnosis were considered. Only uveitis and IBD were analysed as psoriasis predated the majority of PsA diagnoses (∼85%). Only patients with available EMM onset date were included.

Diagnostic delay was categorized by the median, that is, 5 years or more delay in AS and 3 years or more in PsA. An additional sub-analysis using 5 years or more in PsA was also performed. Time-to-event analysis was performed and defined as time from disease diagnosis (AS or PsA) to onset of EMM or to the last available follow-up date and described using Kaplan–Meier plots and log-rank test. Cox proportional hazard analysis was performed adjusting for sex, age of onset and geographical location to assess the association between diagnostic delay on incidence of EMM. The proportional hazards assumption was examined using statistical (Schoenfeld residuals) and graphical approaches. All analysis was conducted using Stata version 16.1 (StataCorp, College Station, TX, USA).

## Results

### Demographic characteristics of cohort populations

Data from 3553 patients were analysed comprising 2097 with AS and 1456 with PsA. Demographic characteristics of the different cohorts are presented for AS and PsA in Table 1. Subjects with AS in Latin America were younger, with correspondingly shorter disease duration, at the time of enrolment (last available follow-up) compared with the Barcelona and Leeds Cohorts. Similar diagnostic delay was observed in the three cohorts.

**Table 1.** Demographic characteristics of AS and PsA patients from the respective geographical cohorts

	Latin America	Barcelona	Leeds	*P*-value
AS^a^				
*n*	1169	472	456	
Age, mean (s.d.), years	45.6 (14.6)	56.5 (15.8)	51.4 (14.5)	<0.001
Sex, male, *n* (%)	867 (74.2)	335 (71.1)	324 (72.8)	0.44
HLA-B27 positive, *n* (%)	–	347 (77.6)	251 (81.8)	0.17
Age at symptom onset, median (IQR), years	26.0 (19.0, 36.0)	25.0 (20.0, 33.0)	24.0 (18.0, 32.0)	<0.001
Disease duration, median (IQR), years	7.0 (4.0, 13.0)	19.5 (10.0, 35.0)	13.5 (7.0, 24.0)	<0.001
Diagnostic delay, median (IQR), years	4.0 (1.0, 10.0)	3.0 (1.0, 8.0)	5.0 (2.0, 10.0)	<0.001
PsA^b^				
*n*	392	442	622	
Age, mean (s.d.), years	53.5 (13.6)	59.6 (14.1)	53.5 (13.2)	<0.001
Sex, male, *n* (%)	201 (51.3)	214 (48.4)	298 (50.6)	0.68
HLA-B27 positive, *n* (%)	–	46 (11.8)	57 (18.8)	0.01
Age at symptom onset, median (IQR), years	42.0 (32.0, 51.0)	41.0 (30.0, 51.0)	38.0 (27.0, 48.0)	<0.001
Disease duration, median (IQR), years	6.0 (3.0, 10.0)	13.0 (7.0, 24.0)	10.0 (6.0, 13.0)	<0.001
Diagnostic delay, median (IQR), years	1.0 (0.0, 5.0)	2.0 (1.0, 6.0)	1.0 (0.0, 2.0)	<0.001
Psoriasis duration, median (IQR), years	16.0 (9.0, 26.0)	29.0 (17.0, 40.0)	22.0 (12.0, 34.0)	<0.001
PsA diagnosed after psoriasis, *n* (%)	203 (85.7)	366 (88.0)	376 (87.9)	0.65
Psoriasis-PsA delay, median (IQR), years	6.0 (1.0, 15.0)	9.0 (2.0, 19.0)	7.0 (1.0, 20.0)	0.026

a Missing data proportion is included in Supplementary Table S1, available at *Rheumatology* online.

b Missing data proportion is included in Supplementary Table S2, available at *Rheumatology* online. IQR: interquartile range.

For PsA, the vast majority of patients presented with PsA after psoriasis with similar proportions in all cohorts. A longer delay between psoriasis and PsA onset was seen in the Barcelona cohort, which also had longer disease duration and older age of onset when compared with the Leeds and Latin America cohorts (Table 1). Missing data proportions of the different variables are shown in Supplementary Tables S1 and S2, available at *Rheumatology* online.

### Overall prevalence of extra-musculoskeletal manifestations by geographical location and diagnosis

**Figure 1.**
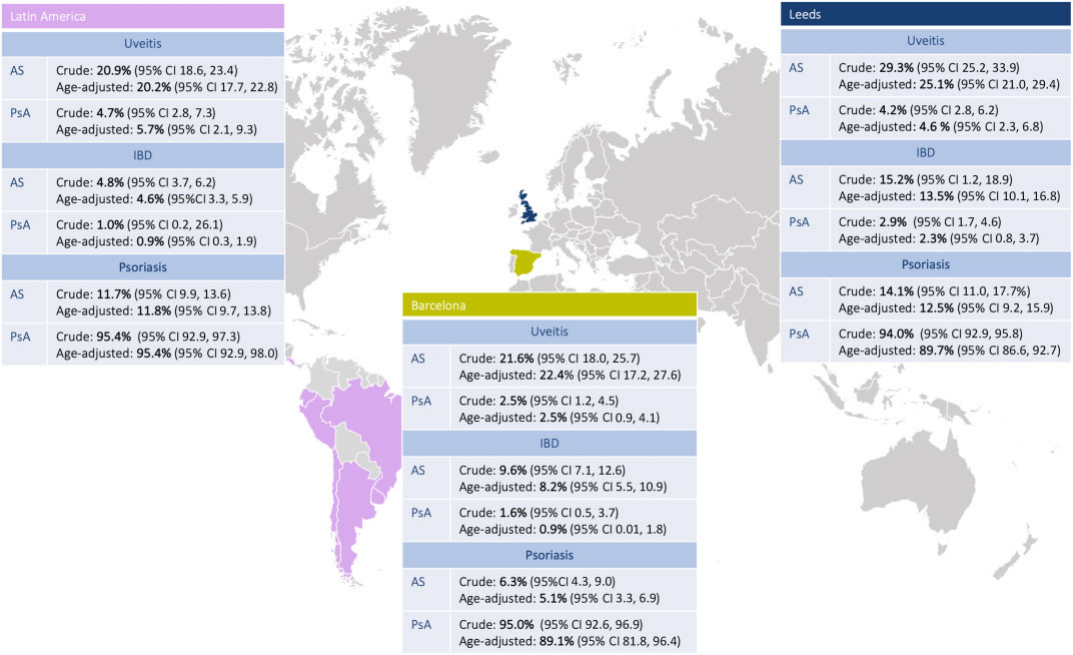
Crude and age-adjusted prevalence of extra-musculoskeletal manifestations divided by location and disease

The overall prevalence of uveitis in the three cohorts was 22.9% (95% CI: 21.1, 24.8) in AS and 3.8% (95% CI: 2.9, 5.0) in PsA; 8.1% (95% CI: 7.0, 9.4) and 2.1% (95% CI: 1.3, 2.9), respectively, for IBD, and 11.0% (95% CI: 9.7, 12.4) and 94.6% (95% CI: 93.0, 95.9), respectively, for psoriasis. Crude and age-standardized prevalence are presented across geographical locations and disease (Fig. 1).

### Relationship between extra-musculoskeletal manifestations onset and SpA diagnosis

Divided by diagnosis, EMMs were more prevalent in the AS population (28.9%, *n* = 600/2073 uveitis and/or IBD *vs* 5.8%, *n* = 74/1284 in the PsA group). For AS patients with uveitis, the first episode of uveitis occurred before AS diagnosis in 45.1%. Of patients with uveitis, 33.3% reported a first episode of uveitis before the diagnosis of PsA. Regarding IBD, 37.4% of AS patients with concomitant IBD were diagnosed with IBD before the AS diagnosis and 70% of patients with PsA and concomitant IBD were diagnosed with IBD before the PsA diagnosis.

### Diagnostic delay and extra-musculoskeletal manifestations

**Figure 2.**
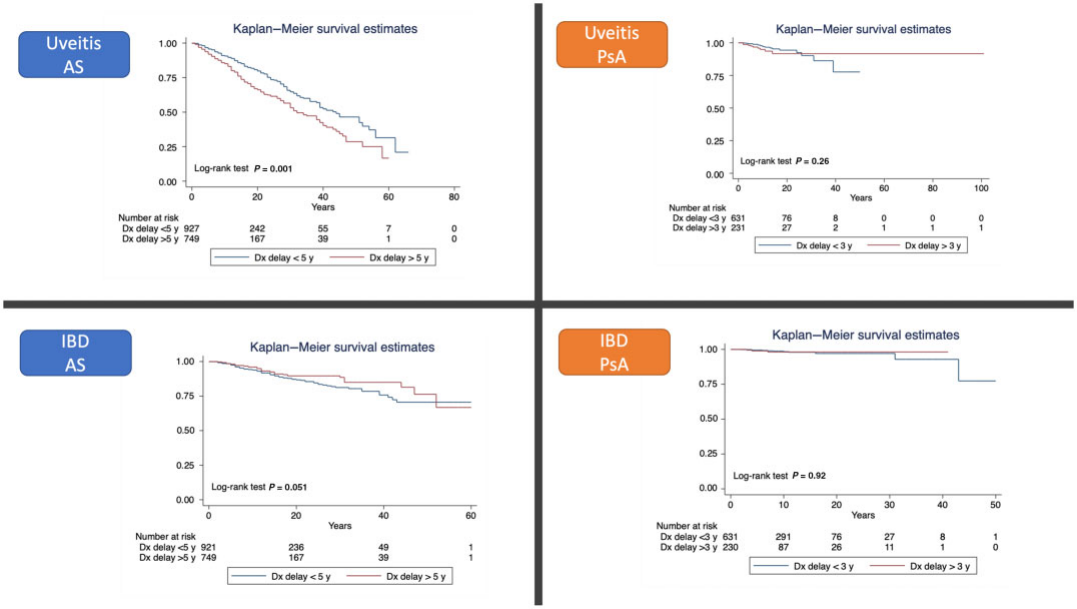
Kaplan–Meier survival curves divided by disease and extra-musculoskeletal manifestation. Dx: Diagnostic; y: years

For the time-to-event analysis, only EMMs that happened after the diagnosis of SpA (AS/PsA) were considered. Demographic variables and their relationship to incidence of uveitis and IBD (incidence rate ratios) after diagnosis of SpA are presented in Supplementary Table S3a and b, available at *Rheumatology* online. Kaplan–Meier graphs are presented for each EMM (uveitis and IBD) divided by diagnosis (AS or PsA) according to diagnostic delay (Fig. 2). In the multivariable Cox model, AS patients with longer diagnostic delay (≥5 years) had more uveitis events (hazard ratio [HR] 4.01; 95% CI: 3.23, 4.07) when adjusted for age of onset, sex and location. This was also observed in AS patients with IBD (HR 1.85; 95% CI: 1.28, 2.67). HRs for diagnostic delay were not significant for uveitis (HR 1.57; 95% CI: 0.69, 3.59) or IBD events (HR 1.59; 95% CI: 0.39, 6.37) in PsA. No differences were seen in additional analyses on PsA patients divided by a 5-year diagnostic delay cut-off (Supplementary Fig. S1, available at *Rheumatology* online).

## Discussion

This study examined the prevalence of EMMs in different SpA (AS and PsA) cohorts and their relationship with diagnostic delay. Our results show that, despite some differences in demographic factors, the prevalence of uveitis and IBD is higher in AS when compared with PsA. Interestingly, when exploring the characteristics of SpA patients with EMMs, we found that a significant proportion presented with uveitis or IBD before the onset of the SpA diagnosis. Furthermore, longer diagnostic delay was associated with a higher probability of uveitis and IBD in AS, suggesting that longer time of uncontrolled inflammation might influence the incidence of EMMs.

In our study, the combined prevalence of uveitis, IBD and psoriasis in AS was 22.9%, 8.1% and 11.0%, respectively, in line with previous publications [4]. Similarly, in PsA, the combined prevalence of uveitis, IBD and psoriasis was 3.8%, 2.1% and 94.6%, respectively, also consistent with results published in a recent meta-analysis [5]. There were some differences across geographic cohorts, such as higher prevalence of uveitis and IBD seen in the Leeds cohort when compared with the other cohorts in this study and a prior AS meta-analysis, which may be explained by a longer disease duration as previously shown [4, 16]. Interestingly, IBD prevalence in AS was significantly lower in the Latin American population compared with the other two cohorts. This is consistent with a recent report of the multi-country observational study PROOF including axSpA patients, outlining an IBD prevalence of just 2.3% in Latin America [17]. Access to prompt secondary care or perhaps differential genetic factors underlying the populations might partly explain this difference when compared with the European cohorts.

Overall, 35% of AS and up to 70% of PsA patients in our study presented with IBD before the SpA diagnosis was made. We decided to exclude these patients in the time-to-event analysis for a number of reasons. Firstly, individuals with a pre-existing history of EMMs are more likely to experience subsequent events or flares, which may confound the results of the analysis. Secondly, longer diagnostic delay may influence the risk of subsequent episodes of flares of EMMs, and excluding patients with prior EMMs can help mitigate this potential confounding factor. Additionally, diagnostic delay was defined as the time from onset of articular (peripheral joint or spinal symptoms) to SpA diagnosis, and thus including patients with pre-existing EMMs may bias the results, as EMMs may have occurred before the onset of joint symptoms. Although our decision to exclude a considerable number of patients may be viewed as a limitation, it is noteworthy that our findings align with previous literature. Specifically, a recent analysis from the Swedish Registry demonstrated that patients with IBD were diagnosed with SpA both before and after their IBD diagnosis. Notably, this association was most pronounced during the 2-year period preceding and following the diagnosis of IBD [18]. Nevertheless, the cumulative incidence of SpA kept increasing in the first 10 years following the diagnosis of IBD particularly in those with Crohn’s disease. In line with our findings, the strongest association in IBD was found with incident PsA (HR 12.0; CI: 10.8, 13.4) outlining that most PsA diagnoses happen after the IBD diagnosis. In the same manner, our data show that in ∼40% of patients, uveitis occurred before the SpA diagnosis. These findings highlight gastroenterology and ophthalmology clinics as potential stages for earlier identification of PsA and axSpA. Indeed, awareness of the need for early referral strategies from ophthalmology to rheumatology is increasing. The SENTINEL study enrolled 798 patients with anterior uveitis that were thoroughly assessed by a rheumatologist finding a prevalence of 50% for axSpA and 17.5% for peripheral SpA [19]. Referral strategies in IBD are also being explored to identify undiagnosed SpA [20]. Yet, our data show that long diagnostic delays remain an unmet need and influence the appearance of uveitis and IBD, which highlights the need for multi-specialty collaborative work at the clinical level in order to improve and enhance referral strategies between ophthalmology, gastroenterology, dermatology and rheumatology.

To our knowledge, this is the first study using data from cohorts across two continents to show that diagnostic delay is associated with a greater probability of uveitis and IBD in AS patients. In line with our findings, Bilge *et al.* used data from a Turkish cohort including rheumatoid arthritis and SpA patients to show that delay to diagnosis was associated with uveitis in the bivariate analysis [10]. Similarly, Gevorgyan *et al.* reported a longer diagnostic delay (10.9 *vs* 5.9 years, *P* < 0.001) when comparing patients with and without uveitis in a single US academic centre [21]. These results, together with our findings, suggest that a longer delay translates into a longer period with uncontrolled inflammation that might influence the appearance of uveitis. Cakar *et al.* found higher CRP levels in AS patients with a longer diagnostic delay, while a recent meta-analysis found higher burden of disease in those with delayed diagnosis including mobility and functional indices [22, 23]. Additionally, Varkas *et al.* found that longer disease duration in axSpA patients was associated with a higher risk of developing anterior uveitis and IBD. Also, higher mean levels of CRP in patients with uveitis and IBD suggest a cumulative exposure to inflammation, although no data on diagnostic delay were reported [24].

Our analysis did not find a significant association between longer diagnostic delay and EMMs in PsA. Small numbers and lower EMM incidence in PsA might be a possible explanation. However, a higher proportion of PsA patients are likely to have received DMARDs for peripheral joint involvement or for psoriasis treatment, which might influence the development of EMMs. Treating cutaneous psoriasis with biologic DMARDs may decrease the incidence of PsA, suggesting that a better control of inflammation may also impact the incidence of EMMs [25]. Unfortunately, treatment data were not available in our cohorts to explore this question. Larger, longitudinal studies with detailed treatment data are needed to confirm this hypothesis.

There were limitations to this study. Patients were enrolled in the study based on their primary clinician diagnosis rather than validated criteria such as the Classification Criteria for Psoriatic Arthritis (CASPAR) or modified New York (mNY) criteria [26, 27]. The characteristics of EMMs, including the time of onset, were either recalled by the participants or extracted from clinical notes, which may have introduced measurement bias. However, this limitation is common where investigators rely on patient recall or clinical notes; prospective designs also have limitations, e.g. attrition and cost. Another limitation is missing HLA-B27 data and lack of treatment data in our cohorts, both of which could potentially impact the incidence of EMMs. Moreover, CRP was not collected systematically in all cohorts, which prevented us from exploring the hypothesis that the effect of diagnostic delay on EMM events is explained by uncontrolled inflammation.

In conclusion, these data show that the prevalence of EMMs including uveitis, IBD and psoriasis is higher in AS than PsA, with a substantial proportion presenting before the onset of the articular disease. A longer diagnostic delay is associated with a greater probability of uveitis and IBD in AS, perhaps due to uncontrolled inflammation over time. These results highlight the need for multi-specialty collaboration to improve diagnostic strategies and referral pathways in order to reduce diagnostic delay in SpA.

## Supplementary Material

kead225_Supplementary_Data

## Data Availability

Data are available upon reasonable request to the corresponding author.
