# Supplementary material for: Diagnostic delay is associated with uveitis and inflammatory bowel disease in AS: a study of extra-musculoskeletal manifestations in SpA
Source: Rheumatology (Oxford). 2023 May 15;63(2):430–5. doi: 10.1093/rheumatology/kead225 (PMC10836992; doi:10.1093/rheumatology/kead225)
Supplement: kead225_Supplementary_Data [file kead225_supplementary_data.pdf]

Supplementary Material. Diagnostic delay is associated with Uveitis and Inflammatory Bowel Disease in AS: a study of extra-musculoskeletal manifestations in SpA.

Supplementary Table 1: Missing data proportions of demographic variables in AS divided by geographical location.

AS	***Iberian America***	***Barcelona***	***Leeds***
N	1169	472	456
Age	3/1169 (0.3%)	0/472 (0%)	1/456 (0.2%)
Sex	0/1169 (0%)	1/472 (0.2%)	11/456 (2.4%)
HLA-B27 positivity	1169/1169 (100%)	25/472 (5.30%)	149/456 (32.7%)
Age symptom onset	32/1169 (2.7%)	10/472 (2.1%)	100/456 (21.9%)
Disease duration	288/1169 (24.6%)	8/472 (1.7%)	50/456 (10.96%)
Diagnostic delay	299/1169 (25.6)	16/472 (3.4%)	111/456 (24.3%)
EMM onset date	145/290 (50%)	35/140 (25%)	93/168 (55.4%)

Supplementary Table 2: Missing data proportions of demographic variables in PsA divided by geographical location.

PsA	***Iberian America***	***Barcelona***	***Leeds***
N	392	442	622
Age	1/392 (0.3%)	1/442 (0.1%)	3/622 (0.5%)
Sex	0/392 (0%)	0/442 (0%)	33/622 (5.3%)
HLAB27	392/392 (100%)	51/442(11.5%)	318/622 (51.1%)
Age symptom onset	12/392 (3.1%)	10/442 (2.3%)	57/622 (9.2%)
Disease duration	67/392 (17.1%)	95/442 (21.5%)	159/622 (25.6%)
Diagnostic delay	70/392 (17.9%)	95/442 (21.5%)	180/622 (28.9%)
Psoriasis duration	155/392 (39.5%)	22/442 (5%)	189/622 (30.4%)

PsA after psoriasis	155/392 (39.5%)	26/442 (5.9%)	194/622 (31.2%)
Psoriasis-PsA delay	189/392 (48.2%)	76/442 (17.2%)	246/622 (39.6%)
EMM onset date	12/21 (57.1%)	5/15 (33.3%)	8/37 (21.6%)

Supplementary Table 3 a and 3b: Incidence rate ratios of demographic characteristics divided by EMM (Uveitis/IBD) and Disease (AS/PSA).

Uveitis	***AS***		***PsA***	
Variables	Incidence Rate Ratio	95% CI	Incidence Rate Ratio	95% CI
Sex (F/M)	1.1	0.88-1.39	1.86	0.83-4.47
Age onset categories				
- 18-29 y	0.95	0.76-1.19	1.71	0.77-3.74
- 30-39 y	1.02	0.78-1.33	0.91	0.33-2.18
- 40-49 y	1.42	0.98-2.02	0.75	0.22-1.99
- 50-59 y	0.48	0.17-1.06	0.90	0.23-2.60
- >60 y	0.45	0.01-2.53	-	-
HLA-B27	**1.99**	**1.17-3.64***	**6.6**	**1.98-21.99***
Diagnostic delay	**1.55**	**1.27-1.92***	1.46	0.61-3.29

IBD	***AS***		***PsA***	
Variables	Incidence Rate Ratio	95% CI	Incidence Rate Ratio	95% CI
Sex (F/M)	**2.61**	**1.86-3.66***	2.28	0.66-9.97
Age onset categories				
- 18-29 y	**0.36**	**0.25-0.51***	0.32	0.03-1.44
- 30-39 y	1.68	1.13-2.46	0.49	0.05-2.20
- 40-49 y	**2.21**	**1.28-3.6***	2.76	0.79-9.06
- 50-59 y	**3.35**	**1.78-5.85***	1.06	0.12-4.78
- >60 y	2.47	0.29-9.11	2.86	0.32-12.9
HLA-B27	**0.21**	**0.14-0.34***	1.06	0.02-8.74
Diagnostic delay	0.70	0.49-1.01	0.84	0.15-3.17

Supplementary Figure 1: Kaplan Meier survival curves of EMMs in PsA divided by diagnostic delay (5 year cut-off)

Uveitis
PsA

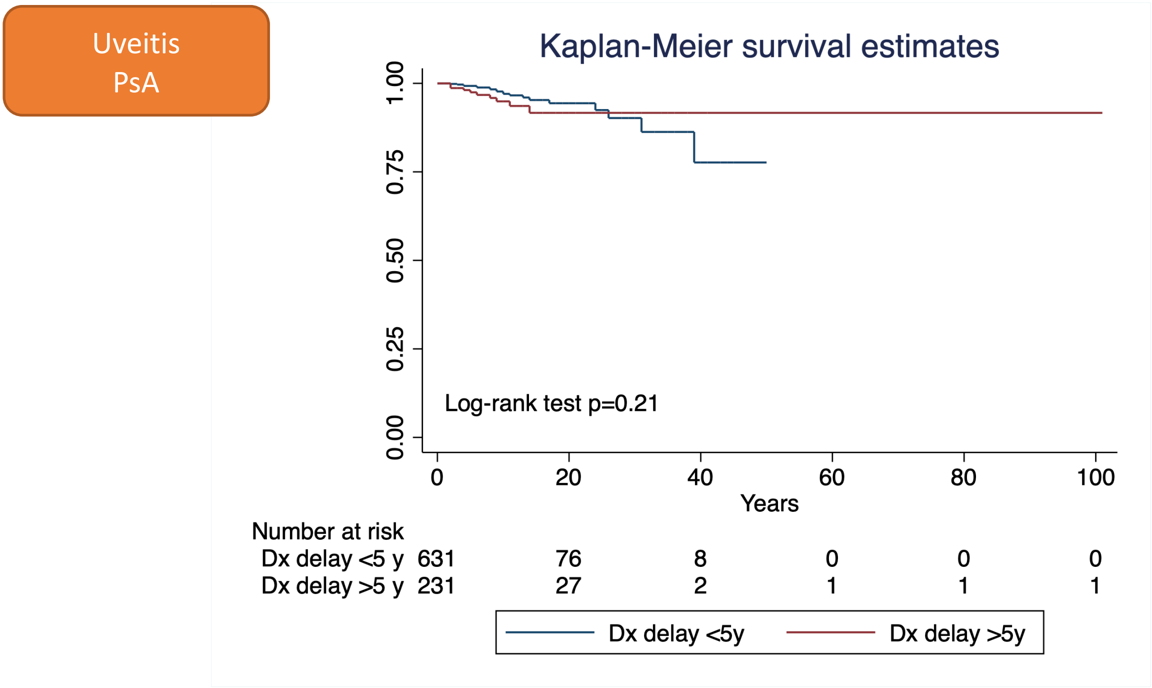

IBD
PsA

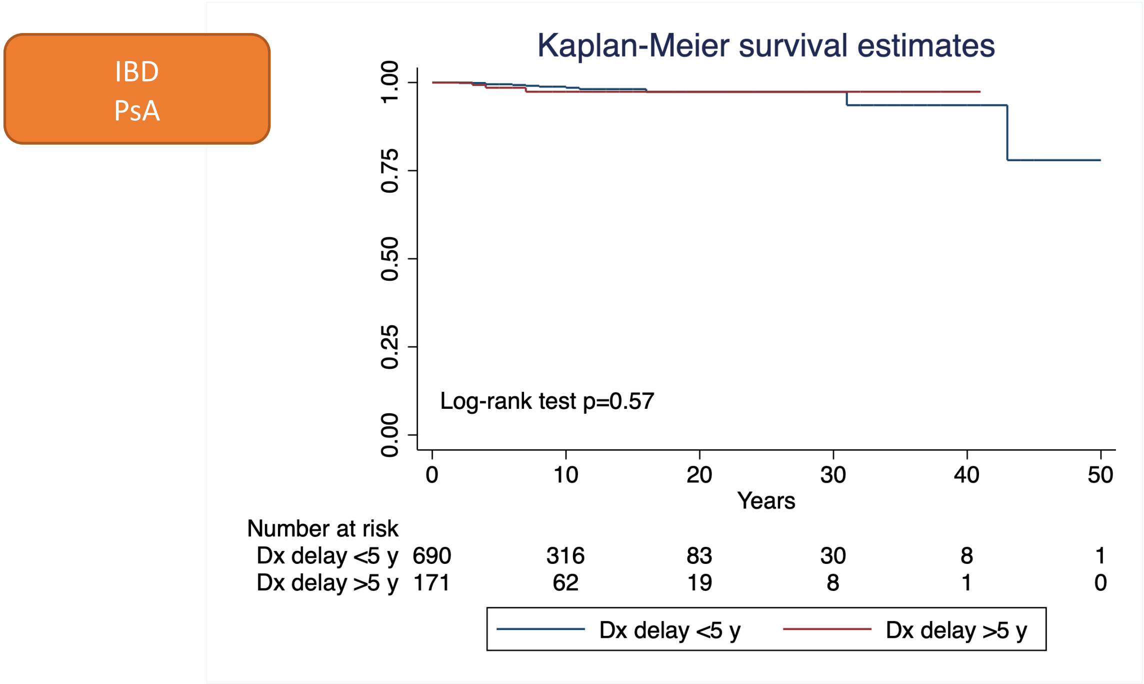

In the multivariable Cox regression analysis, HRs for diagnostic delay divided by a 5 year cut-off were not significant for uveitis (HR 1.58, 95%CI 0.64-3.91) or IBD events (HR 2.73, 95%CI 0.65-11.52) in PsA.
